# The planar cell polarity protein Vangl2 bidirectionally regulates dendritic branching in cultured hippocampal neurons

**DOI:** 10.1186/s13041-014-0079-5

**Published:** 2014-11-12

**Authors:** Akari Hagiwara, Misato Yasumura, Yamato Hida, Eiji Inoue, Toshihisa Ohtsuka

**Affiliations:** Department of Biochemistry, Faculty of Medicine/Graduate School of Medicine, University of Yamanashi, 1110 Shimokato, Chuo, Yamanashi 409-3898 Japan; KAN Research Institute, Hyogo, Japan

**Keywords:** Planar cell polarity signal, Van Gogh-like protein, Dendritic spine, Sholl analysis

## Abstract

**Background:**

Van Gogh-like (Vangl) 2 is a planar cell polarity (PCP) protein that regulates the induction of polarized cellular and tissue morphology during animal development. In the nervous system, the core PCP signaling proteins have been identified to regulate neuronal maturation. In axonal growth cones, the antagonistic interaction of PCP components makes the tips of filopodia sensitive to guidance cues. However, the molecular mechanism by which the PCP signaling regulates spine and dendritic development remains obscure.

**Findings:**

Here we explored the finding that a loss of function of Vangl2 results in a significant reduction in spine density and complexity of dendritic branching. In spite of a previous report, in which the Vangl2 C-terminal TSV motif was shown to be required for the interaction with PSD-95 and the C-terminal intracellular domain was shown to associate with N-cadherin, overexpression of deletion mutants (Vangl2-∆TSV and Vangl2-∆C) had little effect on spine density. However, when an N-terminal region deletion mutant was overexpressed, spine density was slightly down-regulated. Intriguingly, the deletion mutants had a more potent effect on dendritic branching, such that the deletion of the N-terminal region reduced dendritic branching, whereas deletion of the C-terminal region increased it.

**Conclusions:**

Based on these results, Vangl2, a core PCP signaling pathway component, appears to have a functional role in neural complex formation. Especially in the case of dendritic branching, Vangl2 serves as a molecular hub to regulate neural morphology in opposite directions.

## Introduction

One of the most complex and elaborate structures in the brain is the dendritic branches, which are required for precise processing of information coming from a large number of presynaptic inputs [1-3]. Studies using invertebrate and vertebrate systems have revealed that some planar cell polarity (PCP) components affect dendritic morphology and development [4-6]. Van Gogh (Vang), also known as Strabismus (Stbm) was originally identified in *Drosophila* as one of the core PCP proteins, mutations in which caused significant misorientation of organized epithelial structures such as hairs on the wing cells, bristles on the legs, and ommatidia of eyes [7]. *Vang/Stbm* is evolutionarily conserved in many species, and a mammalian homolog *Van Gogh-like (Vangl) 2* has been shown to be expressed in developing and mature mouse brain, at least at the mRNA level [8,9].

In a previous report, we showed that Vangl2 is tightly associated with the postsynaptic density (PSD) fraction and forms a protein complex with PSD-95 and NMDA receptors [10]. Vangl2 directly binds to the third PDZ domain of PSD-95 via its C-terminal TSV motif. This interaction may be required for localization of Vangl2 to synaptic spines [10]. Furthermore, Vangl2 directly binds to N-cadherin, and this interaction may regulate spine formation [11]. However, it is still largely unknown how Vangl2 regulates postsynaptic morphology including spine formation and dendritic branching.

Here, we show that Vangl2 knockdown by shRNA caused significant reductions of spine density and the complexity of dendritic branching. Concerning dendritic complexity, the branching was down- and up-regulated by overexpression of deletion mutants for N- and C-terminal regions of Vangl2, respectively. These results suggest that Vangl2 plays pivotal roles in postsynaptic functions, and may serve as a molecular hub to regulate neural morphology in opposite directions, at least for dendritic branching.

## Results and discussion

### Vangl2 is required for spine and dendritic development in primary cultured neurons

**Figure 1:**
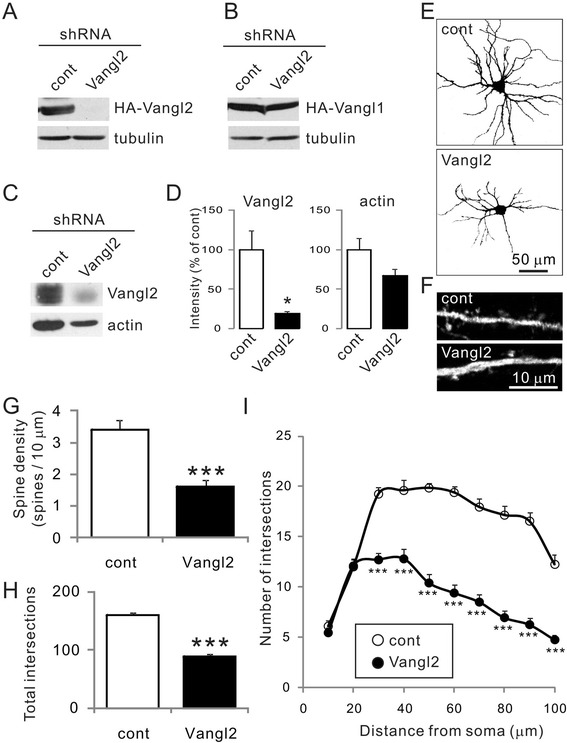
**Effect of Vangl2 knockdown on neuronal morphology. (A, B)** Knockdown (KD) effect of shRNA-Vangl2 on transiently expressed HA-Vangl2 (A) or HA-Vangl1 (B) using the calcium phosphate method in COS-7 cells. The lysate of the cells (20 μl of the lysate each) was analyzed by western blotting using anti-HA and -tubulin antibodies. **(C)** The shRNA-control and -Vangl2 vectors were transfected by nucleofection into neurons at 0 DIV. Endogenous Vangl2 at 8 DIV neurons (10 μg of protein each) was analyzed by western blotting using anti-Vangl2 and -actin antibodies. **(D)** The intensities of protein bands for Vangl2 and actin (n =3 for each band) were analyzed. *p <0.05, Student’s *t*-test. **(E, F)** Cultured hippocampal neurons transfected with control or Vangl2 KD vector were visualized by immunohistochemistry using the anti-GFP antibody. **(G)** The spine density measured at 21 DIV was significantly decreased in Vangl2 KD neurons. Data are means ± SEM (n =10). ***p <0.001, Student’s *t*-test. **(H, I)** The dendritic complexity of neurons transfected with the indicated vectors was measured by Sholl analysis, which shows the number of dendrites crossing circles (vertical axis) at various radial distances from the cell soma (horizontal axis). Intersections at various radial distances (10–100 μm) and total intersections were significantly suppressed by the Vangl2 KD vector. Data are means ± SEM (n =20). ***p <0.001, Student’s *t*-test.

Vertebrates have two *Vangl* genes, *Vangl1* and *Vangl2*, which share ~70% similarity [12]. Its developmental gene expression pattern revealed that *Vangl1* is not strongly related to brain development, but more likely to the development of non-neural elements such as skin and hair bulbs [9]. In contrast, *Vangl2* is strongly expressed in neural structures, and its expression is maintained during the maturation period in some specialized areas such as the cerebellar external granular layer, the dentate gyrus, and the rostral migratory stream [9]. To further understand the role of Vangl2 in neural development, we attempted to knockdown Vangl2 in primary cultured rat hippocampal neurons. The shRNA-mediated knockdown vector for Vangl2 containing GFP [13] could suppress coexpressed HA-Vangl2 in COS-7 cells, but not co-expressed HA-Vangl1 (Figure 1A, B). The knockdown vector inhibited the expression of endogenous Vangl2 in cultured hippocampal neurons (Figure 1C, D). Then, we introduced the knockdown vector into cultured neurons at 4 DIV and examined dendritic branching at 10 DIV (Figure 1E), and spine density at 21 DIV (Figure 1F). In these neurons, we confirmed that the spine density, identified as protrusions with GFP fluorescence, was significantly decreased in neurons treated with a shRNA for Vangl2 compared with neurons treated with a control shRNA (Figure 1G, shRNA-control, 3.4 ± 0.3 spines/10 μm; shRNA-Vangl2, 1.6 ± 0.2 spines/10 μm; n = 10 neurons each, p <0.001 Student’s *t*-test), as previously reported [11]. Moreover, we found that the knockdown of Vangl2 affected developmental dendritic branching at 10 DIV (Figure 1E). To further explore the function of Vangl2, we examined dendritic formation using Sholl analysis, a method that can quantify the number of intersections between the dendritic arbor and a series of concentric circles of increasing radii centered at the cell body (Figure 1H, I) [14,15]. The total amount of intersections was significantly decreased in neurons with Vangl2 knockdown (shRNA-control, 160.1 ± 3.7; shRNA-Vangl2, 89.3 ± 3.9; n =20 neurons each, p <0.001 Student’s *t*-test). These results suggested that Vangl2 regulates not only spine formation, but also dendritic development.

### Effect of Vangl2 deletion mutants on spinogenesis

**Figure 2:**
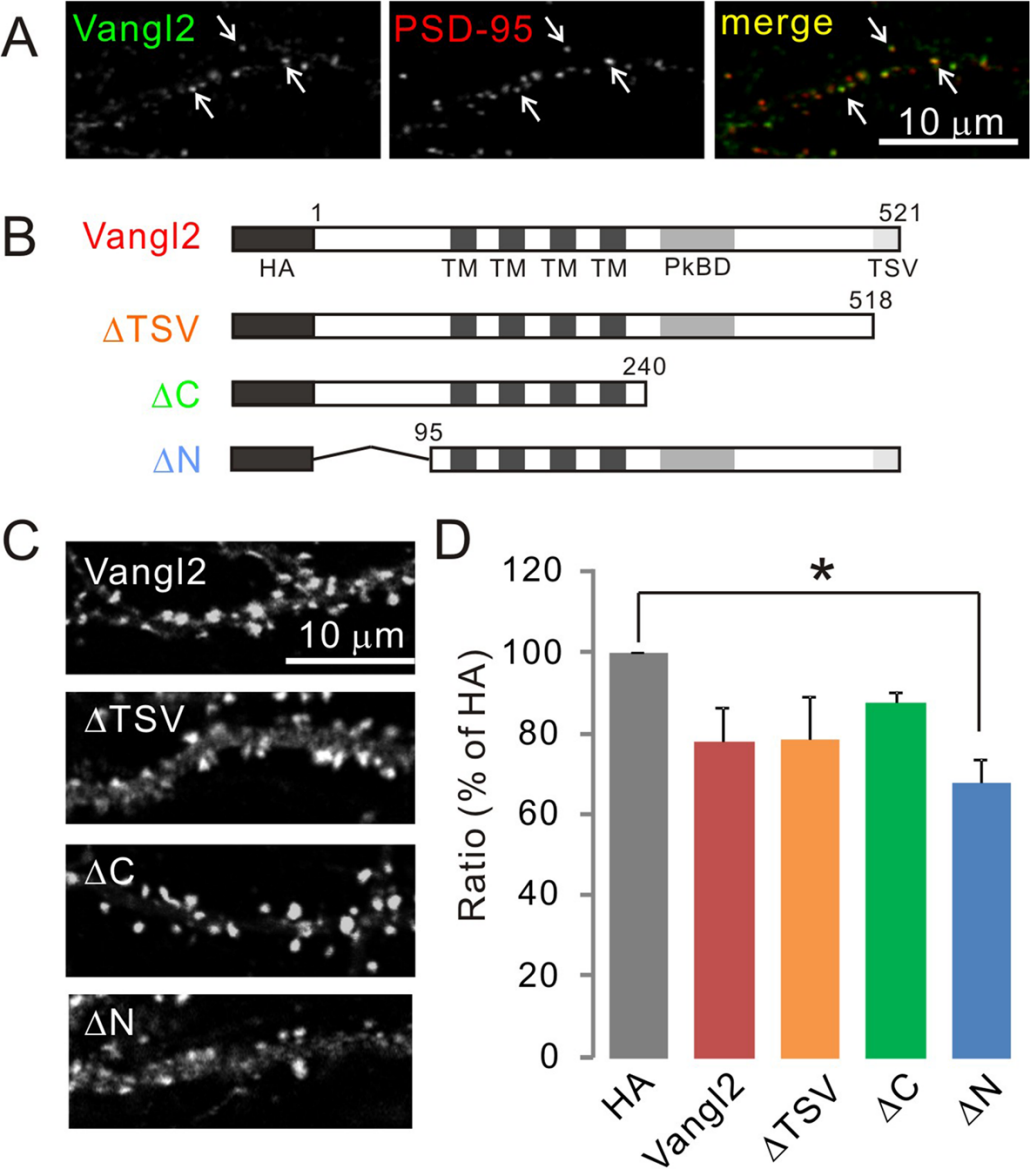
**Effect of various mutants of Vangl2 on spine morphology. (A)** The expression pattern of endogenous Vangl2 in the cultured hippocampal neurons at 21 DIV. Vangl2 was colocalized with PSD-95. **(B)** Schematic representation of HA-tagged Vangl2, and the constructs of deletion mutants. Numbers indicate amino acid sequences. TM, transmembrane region; PkBD, Prickle binding domain. **(C)** Spine morphology. Cultured hippocampal neurons were visualized by immunostaining for pmEGFP-β-actin at 21 DIV. **(D)** Ratio of spine density in neurons transfected with each Vangl2 construct. Data are mean % value relative to the level with control HA vector and SEM from four independent primary cultures. *p <0.05 as assessed by one-way ANOVA.

To address the molecular mechanisms by which Vangl2 regulates spinogenesis, we tested the effects of different deletion mutants (Figure 2B). Vangl2 is a four-pass transmembrane protein with N- and C-terminal domains located in the cytosol. Conserved amino acids (TSV) positioned at the C-terminus promote interactions with PDZ domain-containing proteins [10,12,16]. Analysis of biochemically prepared subcellular fractions from rat brain homogenate revealed Vangl2 as a PSD component, and showed it to be distributed in a punctate pattern colocalizing with PSD-95 (Figure 2A) [10,11]. We previously demonstrated that the TSV motif was necessary for binding to PSD-95 [10]. Furthermore, another PCP component Prickle-binding domain in the C-terminal intracellular domain is required for the interaction of Vangl2 with N-cadherin [11]. Their interaction enhances the internalization of N-cadherin, which may facilitate the flexibility of spine formation. To clarify this, we overexpressed several types of HA-tagged deletion mutants of Vangl2 in cultured hippocampal neurons at 4 DIV, and visualized spine morphology via coexpressed pmEGFP-β-actin at 21 DIV (Figure 2C). Contrary to expectations, the overexpression of Vangl2-ΔTSV and Vangl2-ΔC had little effect on spine development (Table 1, Figure 2D). However, expression of Vangl2-ΔN slightly decreased the spine density (Figure 2D). This result indicated the possibility that Vangl2 regulates spinogenesis via its N-terminal region.

**Table 1:** **Spine density (number / 10 μm)**

	**Density (mean ± SEM)**
HA	7.08 ± 0.99
Vangl2	6.27 ± 1.03
∆TSV	5.24 ± 0.27
∆C	6.21 ± 0.88
∆N	4.70 ± 0.56

Spine density was averaged from four independent primary cultures.

### Bidirectional regulation of dendritic branching with Vangl2 mutants

**Figure 3:**
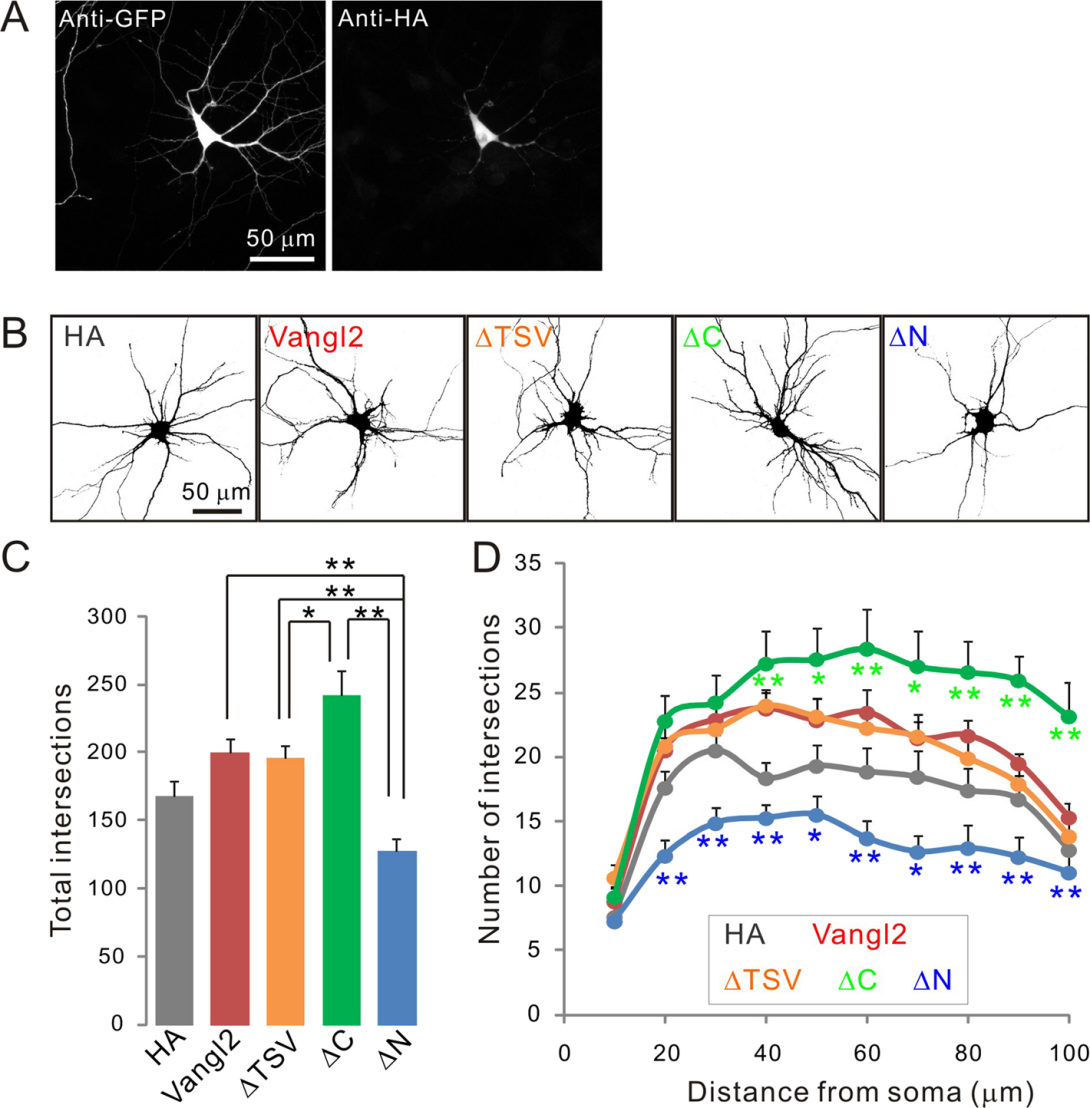
**Effect of Vangl2 on dendrite branching. (A)** Cultured hippocampal neurons were transfected with various HA-tagged constructs of Vangl2 and EGFP at 4 DIV. Neurons were fixed and immunostained using anti-GFP and -HA antibodies at 10 DIV. **(B)** Neurons transfected with Vangl2 constructs were visualized by immunohistochemistry to GFP and the morphology of single neuron was extracted on ImageJ for subsequent Sholl analysis. **(C, D)** The dendritic complexity of neurons expressing the indicated constructs was measured by Sholl analysis. Total intersections and that at various radial distances (10–100 μm) from the center of the cell soma were significantly suppressed by the Vangl2-∆N construct. On the other hand, neurons expressing Vangl2-∆C showed increased dendritic complexity. Data are means ± SEM (n =20). *p <0.05 and **p <0.01 as assessed by one-way ANOVA.

To determine the role of Vangl2 in the expression of complex neural connectivity, we next evaluated dendritic branching by Sholl analysis of neurons transfected with both HA-tagged deletion mutants and GFP (Figure 3A). The overexpression of Vangl2 and Vangl2-ΔTSV slightly increased dendritic complexity compared with overexpression of control HA only, but the total amounts of intersections were not significantly different: HA control, 167.6 ± 11.0; Vangl2, 200.2 ± 9.4, p = 0.3; Vangl2-ΔTSV, 196.3 ± 8.8, p = 0.4 (one way ANOVA, Tukey’s HSD test, n = 20 from two independent primary cultures, Figure 3B-D). Among the Vangl2 mutants, neurons expressing Vangl2-ΔC showed significantly increased branching, and the total number of intersections was 1.21-fold higher than that in neurons expressing full-length Vangl2 (Figure 3B-D, Vangl2-ΔC, 242.2 ± 18.4, n = 20). Expression of a C-terminal intracellular domain deletion mutant had little effect on spine density, but significantly increased dendritic development. This result indicates that there are two different pathways regulating spine and dendritic formation related to Vangl2. Intriguingly, overexpression of Vangl2-ΔN significantly decreased the complexity, and the total amount of intersections was 0.63-fold less than that observed with overexpression of full-length Vangl2 (Figure 3B-D, Vangl2-ΔN, 127.8 ± 8.9, n = 20). The N-terminal intracellular domain of Vangl2 may have key roles in facilitating spine and dendritic development.

We have two hypotheses for how Vangl2 mediates regulation of dendritic branching. First, Vangl2 binds to either an accelerator at the N-terminus (AC-Vangl2) or an inhibitor at the C-terminus (Vangl2-IN). During development, the AC-Vangl2 complex may be prevalent, and the relatively dominant AC-Vangl2-mediated pathway facilitates dendritic branching. With the expression of full length Vangl2 or Vangl2-ΔTSV, the ratio between the AC-Vangl2 and Vangl2-IN complexes remains unchanged, thus having no effect on dendritic branching. However, the expression of Vangl2-ΔN increases the presence of Vangl2-IN, enhancing the inhibitory pathway. On the other hand, expression of Vangl2-ΔC increases the level of the AC-Vangl2 complex, and the enhanced accelerator-mediated signal further facilitates branching. Our second hypothesis is that Vangl2 has an inhibitor at the N-terminus and an accelerator at the C-terminus. Similar to the first hypothesis, expression of Vangl2 or Vangl2-ΔTSV keeps the balance. However, if overexpressed Vangl2-ΔC acts as a dominant active signal to endogenous Vangl2, the inhibitor should be captured by overexpressed Vangl2-ΔC and the accelerator signal on Vangl2 is relatively enhanced. On the other hand, overexpressed Vangl2-ΔN works in a dominant negative fashion, and the inhibitor signal on Vangl2 may suppress branching. Currently, it is unclear which model is the case. Thus, to reveal the molecular mechanism underlying Vangl2-mediated bidirectional regulation of neural development, we need to identify the binding partners of the N- and C-terminal regions of Vangl2 in future research.

## Conclusions

Planar cell polarity has been highly scrutinized in *Drosophila*. In recent years, evidence has accumulated suggesting that the PCP pathway is highly conserved and regulates polarized cellular and tissue morphology. Here, we show a requirement of Vangl2 for spine and dendritic development. N- and C-terminal intracellular domain deletion mutants regulated the dendritic branching bidirectionally, suggesting that Vangl2 is a molecular hub for neural development.

## Materials and methods

### Constructs

Expression vectors for Vangl2 were constructed in pCAII-HA by standard molecular biological methods. Constructs for Vangl2 knockdown were created using the pSUPER vector system (shRNA, pSUPER-neo + GFP, Oligoengine). Sequence of constructs was as follows: control shRNA (5′-GAAACGGAAAGCAGGTACG-3′), rat Vangl2 shRNA (5′-GGGAGAAACAACAACGGTG −3′, [13]).

### Antibodies

We used the following primary antibodies: anti-PSD-95 (1:500, Thermo Scientific), anti-Vangl2 (1:100, [10]), anti-GFP (1:500, Life Technologies), anti-HA (1:400, Roche Applied Science), anti-tubulin (1:1000, Oncogene), and anti-actin (1:500, Merck Millipore). To detect the immunofluorescence signal, Alexa Fluor 488- or 568-labeled secondary antibodies (1:500, Molecular Probes) were used. For western blotting, HRP-linked secondary antibodies (1:2000–5000, GE Healthcare) were used.

### Hippocampal primary culture, transfection, and immunohistochemistry

The following procedures were reviewed and approved by the animal welfare committees at the University of Yamanashi. Primary cultures of hippocampal neurons were prepared from eighteen day old pregnant Wistar rats as described previously [17,18]. Neurons were transfected with shRNA constructs or various mutants of HA-Vangl2 with pmEGFP-β-actin (kindly provided by Dr. H. Bito, University of Tokyo) or EGFP at 4 DIV using Lipofectamine 2000 (Life Technologies). Immunostaining was performed at 10 or 21 DIV. The transfected cells were fixed with 4% paraformaldehyde and treated with blocking solution containing 4% Block Ace (Snow Brand Milk Products), 2% normal goat serum, and 0.2% Triton X-100, in PBS. Then, cells were incubated with primary antibodies diluted in blocking solution for 1 h, followed by secondary antibodies.

### Image acquisition and quantification

Fluorescence images were acquired by confocal laser microscopy (Fluoview FV1000, Olympus) using a 60× oil immersion objective lens. For analysis of spine density, dendritic spines visualized by pmEGFP-β-actin were counted on the main dendritic shaft. For Sholl analysis, neurons labeled with EGFP were visualized in a series of images taken throughout the z aspect of each cell. The number of dendrites crossing each concentric circle with 10 μm differences in diameter around the cell soma was counted using the ImageJ plugin ShollAnalysis (v1.0, provided by the Ghosh Lab, University of California, San Diego). Acquisition of microscopy images and morphometric quantification were performed by investigators blind to the experimental condition.

## Abbreviations

DIV: Day in vitro
NMDA: N-methyl-D-aspartate
PCP: Planar cell polarity
PSD: Postsynaptic density
Stbm: Strabismus
TM: Transmembrane region
PkBD: Prickle binding domain
Vang: Van Gogh
Vangl: Van Gogh-like
